# Rationale and clinical applications of 4D flow cardiovascular magnetic resonance in assessment of valvular heart disease: a comprehensive review

**DOI:** 10.1186/s12968-022-00882-0

**Published:** 2022-08-22

**Authors:** Miroslawa Gorecka, Malenka M. Bissell, David M. Higgins, Pankaj Garg, Sven Plein, John P. Greenwood

**Affiliations:** 1 Leeds Institute of Cardiovascular and Metabolic Medicine, University of Leeds, Leeds, LS2 9JT UK; 2 Philips, Farnborough, England UK; 3 Norwich Medical School, University of East Anglia, Norwich, UK

**Keywords:** 4D flow MRI, 4D flow CMR, Magnetic resonance imaging, Valvular heart disease

## Abstract

**Background:**

Accurate evaluation of valvular pathology is crucial in the timing of surgical intervention. Whilst transthoracic echocardiography is widely available and routinely used in the assessment of valvular heart disease, it is bound by several limitations. Although cardiovascular magnetic resonance (CMR) imaging can overcome many of the challenges encountered by echocardiography, it also has a number of limitations.

**Main text:**

4D Flow CMR is a novel technique, which allows time-resolved, 3-dimensional imaging. It enables visualisation and direct quantification of flow and peak velocities of all valves simultaneously in one simple acquisition, without any geometric assumptions. It also has the unique ability to measure advanced haemodynamic parameters such as turbulent kinetic energy, viscous energy loss rate and wall shear stress, which may add further diagnostic and prognostic information. Although 4D Flow CMR acquisition can take 5–10 min, emerging acceleration techniques can significantly reduce scan times, making 4D Flow CMR applicable in contemporary clinical practice.

**Conclusion:**

4D Flow CMR is an emerging CMR technique, which has the potential to become the new reference-standard method for the evaluation of valvular lesions. In this review, we describe the clinical applications, advantages and disadvantages of 4D Flow CMR in the assessment of valvular heart disease.

## Background

Transthoracic echocardiography (TTE) is the first line imaging modality for assessing patients with valvular heart disease (VHD) [1–3]. Although it is easily accessible, safe and inexpensive [4–6], it may be limited in cases of poor acoustic windows secondary to large body habitus [7] or in the presence of eccentric [8] or multiple regurgitant jets [9]. TTE assessment of heart valve function can also be challenging due to limited reproducibility [9], operator-dependence [10] and inaccuracy in quantification of regurgitant lesions in certain cases [11]. Although transoesophageal echocardiography overcomes some of these limitations and can be performed in patients with suboptimal quality TTE images [1], it is a moderately invasive procedure, can lead to potentially serious complications, [12] and increased patient discomfort.

Phase contrast magnetic resonance (PCMR) imaging offers several advantages over TTE and can clarify the severity and mechanism of VHD lesions in selected cases [7, 8, 13]. It enables detailed assessment of valvular flow and function with no geometric assumptions, and can therefore accurately assess lesions with multiple regurgitant jets or eccentric jets [8, 13]. Furthermore, assessment of left ventricular (LV) function and remodelling by cardiovascular magnetic resonance (CMR) imaging has been shown to be highly accurate and reproducible [14]. CMR is also the reference standard for evaluation of right ventricular (RV) morphology and function, and therefore can precisely assess the impact of right-sided valvular lesions on the ventricle [15]. However, despite all of its advantages, PCMR does not allow for accurate direct jet quantification in the atrio-ventricular valves as it does not account for valve plane motion during the cardiac cycle [13]. Also, flow quantification can be significantly affected by phase-offset errors [7, 16]. Furthermore, errors introduced in ventricular stroke volume calculations can lead to inaccurate quantification of atrio-ventricular regurgitant lesions [6].

Four-dimensional (4D) Flow CMR is a relatively novel CMR technique, which offers time-resolved 3-dimensional (3D) imaging and allows accurate and precise assessment of VHD. It overcomes a lot of the limitations present in TTE and PCMR. A typical 4D Flow whole heart acquisition has a temporal resolution of 30–40 ms, spatial resolution of < 3 mm × 3 mm × 3 mm and takes 5–10 min [16]. Available acceleration techniques, such as kt broad linear speed up technique (*kt* BLAST) with a 32-channel coil array allow shorter scan times, and make 4D Flow CMR more clinically applicable [17]. New acceleration techniques such as prospective undersampling in multiple dimensions (PROUD) [18], k-adaptive-t autocalibrating reconstruction for cartesian sampling (kat-ARC) [19] and *SmartSpeed (*compressed sensitivity encoding featuring artificial intelligence algorithm) [20] are emerging and hold promise for an even faster acquisition. Recommended sequence parameters for 4D Flow CMR imaging are based on the delicate balance between the ideal parameters to provide high quality data and what is clinically feasible in terms of, mainly scan time. The typical sequence parameters are as follows: field of view, which is sufficient to cover the region of interest; k-space segmentation factor of 2, which reduces scan time, but also decreases accuracy; retrospective electrocardiogram (ECG) gating, which allows coverage of the entire cardiac cycle, but requires complex reconstruction—this is preferred to prospective gating, where the data from end-diastole are not acquired, thus impairing accuracy of mitral valve and tricuspid valve forward flow quantification [21]; the use of respiratory navigator; elliptical k-space to reduce scan time; flip angle equivalent to at least the Ernst angle to provide optimal signal-to-noise ratio (SNR), but with negative effect on contrast; the use of acceleration techniques, if available, to reduce scan time, although this benefit is offset by reduction in signal-to-noise ratio; single velocity-encoding (VENC) set to 10% above the expected maximum velocity, which decreases scan time, but also negatively affects the velocity-to-noise ratio; and the application of phase-unwrapping algorithms as well as eddy current and Maxwell correction algorithms to improve accuracy [16].

When compared to standard imaging modalities, such as TTE and PCMR, 4D Flow CMR offers a number of advantages. It enables direct jet visualisation and quantification of regurgitant lesions, especially when complicated by eccentric or multiple jets [22–24]. This is possible as it can be visualised in a 3D dataset. When performed as a whole heart acquisition, it allows the evaluation of all four cardiac valves simultaneously within a single acquisition [25]. Simultaneous quantification of flow across all 4 valves in 4D Flow CMR provides a means of internal validation of flow measurements [16]. Studies which evaluated all four valves, showed strong agreement between net flow volumes across all valves [25], with small inter-valvular variation [26]. Consistency across modalities is significantly improved when retrospective valve tracking is used [27]. Furthermore, as all the measurements are obtained from the same acquisition, the impact of variability related to changes in heart rate, on consistency of measurements will be reduced [28]. Although blurring can occur as a result of irregular heart rate [29], recent study showed that measurements of flow volumes remain accurate and feasible even in the presence of atrial fibrillation [30]. Moreover, peak velocity measurements in stenotic lesions may be more accurate and precise than with PCMR [31]. Several novel markers, such as wall shear stress can also be measured, which may be helpful in the assessment of patients with bicuspid aortic valve (BAV) disease amongst other pathologies [32–35]. Finally, the acquisition itself requires only very simple planning [29].

There are, however, a number of challenges encountered in 4D Flow CMR. The main drawbacks of this technique include limited temporal and spatial resolution, long scan time [16] and the requirement for complex post-processing, which requires specialised knowledge and is time-consuming. Also, 4D Flow CMR imaging requires supplementary cine images, which serve as an anatomical framework for the phase images. This can potentially lead to misalignment between the anatomical reference and the phase images due to heart rate variability and patient movement during the scan. Although misalignment can often be corrected during post-processing, it adds an extra step to the analysis [28].

As with PCMR, appropriate VENC needs to be chosen and should be set to a value that is marginally higher (about 10%) than expected peak velocity in the region of interest. Inappropriately low VENC setting can lead to aliasing, whereas the higher the VENC, the lower the velocity-to-noise ratio. It is therefore advisable to apply a phase-unwrapping algorithm, especially in cases where it might be difficult to estimate the maximum velocity [16]. Mixed VHD can pose a further challenge to VENC setting, as a value appropriate for low velocities will not be optimal for a high velocity setting. Although this can be overcome by two separate acquisitions with different VENC settings, it is very time consuming [36]. Hence, clinically applicable dual- and multi-VENC sequences are emerging, which will be helpful when stenotic and regurgitant lesions co-exist [16, 36, 37].

Although peak velocity assessment in stenotic lesions may be complicated by signal dephasing secondary to turbulent flow and could potentially lead to imprecise measurements, the visualisation and identification of the highest velocity area may also allow more accurate assessment of velocity [38].

With regard to regurgitant lesions, visualisation of areas of turbulence and signal dephasing can help with accurate quantification, as these areas can be avoided [9]. Regurgitant lesions are also frequently complicated by multiple and/or eccentric jets, especially in the case of mitral valve [39]. Although direct jet quantification can be performed in these cases, it may be challenging [40] and time-consuming [41]. The indirect method can be advantageous in these cases and has been shown to have a better intra- and inter-technique reliability [42].

Furthermore, as with PCMR, phase offset errors may occur and must be corrected. Artefacts occurring due to Maxwell terms and non-linearity of the gradient field tend to be optimised easily, however, correction for eddy currents has to be performed manually and incorporated into data analysis [43]. The advantages and disadvantages of the various imaging modalities used in VHD assessment are shown in Table 1.

**Table 1:** Advantages and disadvantages of echocardiography and cardiovascular magnetic resonance in assessment of valvular heart disease

Modality	Advantages	Disadvantages
Transthoracic echocardiography (TTE)	Widely available [6] Inexpensive [5] Safe [4]	Limited accuracy in patients with large body habitus and chronic obstructive pulmonary disease [7] Limited accuracy in the presence of eccentric/multiple regurgitant jets [9] Suboptimal assessment of right heart [8]
Transoesophageal echocardiography	Not limited by body habitus [44] Superior image quality when TTE is suboptimal [1] Visualisation of structures not assessed by TTE e.g. left atrial appendage [1]	Moderately invasive. Risk of bleeding and oesophageal perforation [44] Requires presence of trained medical personnel [44] Potential complications of sedation [44] Reduced utility during pandemic due to high aerosol production [45]
Standard CMR (LV/RV cine stack, PCMR and LGE)	Reference-standard left and right ventricular size and function assessment [14, 15] Accurate indirect quantification of atrio-ventricular valve regurgitation, even in the presence of eccentric and multiple jets [39] Tissue phenotyping/quantification of fibrosis [46]	Inaccurate direct quantification of atrio-ventricular valvular regurgitation [13] Potential for error in stroke volume calculation [6] Limited by claustrophobia/arrhythmia [6, 47]
4D flow CMR	Regurgitant jet visualisation [22] Direct regurgitant jet quantification [9] No geometric assumptions [22] Simultaneous analysis of flow across all four valves [25] Accurate peak velocity assessment vs. PCMR [31] May be advantageous in combined valve lesions [22] Measurement of fluid biomechanics [29] Simple acquisition [29] Free-breathing [23] Plane reformatting is possible [25]	Time-consuming post-processing [29] Limited temporal and spatial resolution [29] Limited software availability [16]

CMR = cardiovascular magnetic resonance; LGE = late gadolinium enhancement; LV = left ventricle; PCMR = phase contrast magnetic resonance; RV = right ventricle; TTE = transthoracic echocardiography

A number of cardiovascular 4D Flow CMR reviews were published in recent years. These focused on different aspects of 4D Flow CMR, such as kinetic energy assessment of LV blood flow [48], technical aspects of 4D Flow acquisition and application of 4D Flow CMR in various cardiac and vascular pathologies [49, 50], congenital heart disease applications [51] as well as structural heart disease [52] and peak velocity assessment [53]. Other reviews evaluated 4D Flow CMR in mitral valve disease [23, 54], left-sided VHD [22] and in various cardiovascular pathologies with a limited focus on VHD [32, 38, 55, 56]. Although a recent systematic review of 4D Flow CMR in the heart and great vessels evaluated current evidence for 4D Flow CMR in VHD in great detail, it did not provide illustrative examples of the different pathologies [27]. To our knowledge, there is no clinically focused 4D Flow CMR review, which is solely dedicated to acquired valvular heart disease.

As acquired VHD was not the emphasis of the majority of the above publications, the purpose of this review is to provide an educational overview of the rationale for 4D Flow CMR, its utility in the assessment of VHD in contemporary clinical practice and to provide illustrative examples of 4D Flow CMR evaluation of common valvular pathologies. Our aim was to provide a clinically focused and illustrative summary of 4D Flow CMR applications in VHD, in a manner that is approachable to both, a beginner 4D Flow CMR imager and an advanced specialist looking for a comprehensive summary of data.

### Rationale and clinical applications of 4D Flow CMR in valve-specific pathologies

#### Mitral regurgitation

Mitral regurgitation and aortic stenosis (AS) are the most common valvular pathologies in the developed world. As the population is ageing significantly, the prevalence of VHD is expected to increase [57]. Mitral regurgitation accounts for almost 25% of VHD cases in contemporary practice and is the second most common pathology in Europe [58]. Furthermore, untreated severe mitral regurgitation is associated with a high burden of morbidity and mortality [59]. Current guidelines recommend surgery in symptomatic patients with severe chronic primary mitral regurgitation and those who are asymptomatic, but have evidence of LV or left atrial (LA) remodelling, such as impaired LV ejection fraction (LVEF) ≤ 60%, LV end-systolic diameter ≥ 45 mm, new-onset atrial fibrillation or pulmonary artery systolic pressures > 50 mmHg. In secondary mitral regurgitation, surgery is recommended in those with LVEF > 30% undergoing coronary artery bypass grafting, those with LVEF < 30%, but with viable myocardium and an option for revascularisation, and those who are considered at low surgical risk and failed a trial of optimal medical therapy [1]. These guidelines highlight the importance of accurate assessment of mitral regurgitation severity and LV cavity size and function, to guide surgical therapy decisions.

Quantification of mitral regurgitation, and therefore the indication for surgery or percutaneous intervention relies on TTE measurements in the majority of patients [1, 3]. In selected cases, however, further investigation, including the use of CMR may be indicated [3, 40, 60, 61]. CMR studies showed that PCMR reclassified a proportion of patients with mitral regurgitation into a different severity category, which in turn showed better association with prognosis. Uretsky et al. in 2015 demonstrated discordance between mitral regurgitation quantification by PCMR and TTE and showed that CMR had a superior correlation with post-operative LV remodelling [62]. The prognostic advantage of CMR was confirmed in several other studies [63–65]. However, studies have reported different thresholds for classifying ‘severe’ mitral regurgitation. A study by Myerson et al., showed that regurgitant volume (RVol) of more than 55 ml and regurgitant fraction (RF) of more than 40% was associated with adverse clinical outcomes [63], whereas a study by Aplin et al. proposed lower threshold values [66].

The above studies utilised indirect techniques for quantification of RVol and RF. This was performed by subtracting aortic forward flow volume (obtained from through-plane velocity mapping) from LV stroke volume (SV) derived from cine images. This decreased errors that could occur as a result of an eccentric jet, multiple jets or flow turbulence leading to signal void [6]. Although this standard PCMR assessment of mitral regurgitation is robust, it requires 2 types of acquisition and its accuracy may be limited by errors introduced in the process of SV calculation [9]. Furthermore, different centres may use different methods of LV segmentation including or excluding LV outflow tract (LVOT) [67] and/or papillary muscles [68], which can lead to discrepancy in mitral regurgitation quantification. In the absence of tricuspid regurgitation (TR), it is also possible to quantify mitral regurgitation by subtracting RV SV from LV SV [6], although this is less commonly used. Direct assessment of the regurgitant flow by PCMR is not typically performed in clinical practice as it is inaccurate due to through-plane motion of the valve plane during systole, which can lead to significant quantitation errors [68]. This is further challenged by the mitral valve’s complex anatomy [22]. Similarly to TTE, in the presence of eccentric jets, quantitation of mitral regurgitation by the direct approach may be imprecise due to signal void [9].

4D Flow CMR overcomes a lot of these limitations and offers several advantages in the assessment of mitral regurgitation [9, 54]. A summary of the main studies that evaluated 4D Flow CMR in the setting of mitral regurgitation and other valvular pathologies is presented in Table 2.

**Table 2:** 4D flow CMR studies in valvular heart disease

Valve pathology	Study	Nr of patients (n)	Population assessed	Reproducibility assessment	4D Flow CMR vs. TTE	4D Flow CMR vs. PCMR	Main findings
Mitral regurgitation	2008 Westenberg et al. [69]	Controls n = 10 Patients n = 20	Ischaemic cardiomyopathy with mitral regurgitation and/or TR	+	−	+	PCMR overestimated transmitral flow in healthy volunteers 4D Flow CMR showed strong agreement between MV and TV flow in patients with mitral regurgitation and/or TR
	2009 Roes et al. [25]	Controls n = 22 Patients n = 29	Ischaemic cardiomyopathy with valvular regurgitation	+	−	−	Agreement amongst net flow volume for all valves was excellent Good intra- and inter-observer reliability for quantification of RF
	2009 Marsan et al. [72]	Patients n = 64	Functional mitral regurgitation	−	+ (3D TTE)	−	2D TTE significantly underestimated mitral regurgitation
	2011 Brandts et al. [71]	Patients n = 47	Ischaemic heart failure	−	+	+	Higher mitral regurgitant fraction vs. PCMR Strong correlation between 4D Flow CMR and TTE for LV diastolic assessment
	2018 Gorodisky et al. [74]	Patients n = 27	Isolated mitral regurgitation of various severity	+	+	+	CMR 4D-PISA was feasible CMR 4D-PISA was smaller than TTE-PISA
	2018 Feneis et al. [9]	Patients n = 21	Isolated mitral regurgitation n = 10 Mitral regurgitation + TR n = 5 Isolated TR = 6	+	−	+	Good correlation between PCMR and 4D Flow CMR quantification of regurgitation by direct and indirect methods
	2019 Kamphuis et al. [70]	Controls = 46 Patients n = 114	Acquired and congenital pathologies	+	−	−	Automated valve tracking is performed more rapidly than manual valve tracking Strong intra- and inter-observer correlation for regurgitant fraction quantification by automated valve tracking
	2020 Blanken et al. [73]	Patients n = 30	Various degrees of mitral regurgitation severity	+	−	+	Valve tracking underestimated mitral regurgitation severity in cases of severe mitral regurgitation SFT RV correlated better with indirect quantification of RV by PCMR than RVT
	2021 Fidock et al. [54]	Patients n = 35	Primary mitral regurgitation n = 12 Secondary mitral regurgitation n = 10 MVR n = 13	+	−	+	Highest reproducibility was found for MV inflow-AV outflow method of mitral regurgitation quantification Good correlation between all methods in secondary mitral regurgitation and MVR
	2021 Spampinato et al. [42]	Controls = 6 Patients = 54	Mitral valve prolapse	+	+	+	Indirect 4D Flow CMR assessment of mitral regurgitation in MVP showed better intra- and inter-technique concordance than direct assessment
	2021 Juffermans et al. [26]	Patients n = 64 Controls n = 76	Various pathologies	+	−	−	Strong-to-excellent interobserver reliability for forward flow volume and net forward volume for all valves Moderate-to-excellent reliability for assessment of RF for all valves
Aortic regurgitation	2013 Ewe et al. [86]	Patients n = 32	Various degrees of AR severity	−	+ (3D TTE)	−	High concordance between 3D-TTE and 4D Flow CMR
	2016 Chelu et al. [85]	Patients n = 54	Various pathologies	−	+	−	AR severity by 4D Flow CMR correlated well with TTE
	2020 Alvarez et al. [79]	Patients n = 34	AR > 5%	−	−	+	AV forward and regurgitant flow by 4D Flow CMR agreed well with PCMR
	2021 Juffermans et al. [26]	Patients = 64 Controls = 76	Various pathologies	+	−	−	Strong-to-excellent interobserver reliability for forward flow volume and net forward volume for all valves Moderate-to-excellent reliability for assessment of RF for all valves
Aortic Stenosis	2013 Dyverfeldt et al. [93]	Controls n = 4 Patients n = 14	Aortic dilatation present in some cases	−	+	−	Patients with AS demonstrated much higher peak total TKE in the ascending aorta
	2014 Garcia et al. [90]	Controls n = 10 Patients n = 40	Tricuspid and bicuspid AS	+	−	+	EOA measurement by 4D Flow CMR jet shear layer detection method was feasible 3D projection of vena contracta by 4D Flow CMR enabled more accurate localisation of the measurement plane
	2016 Negahdar et al. [92]	Controls n = 5 Patients n = 4	≤ moderate AS	−	+	−	Spiral 4D Flow readout resulted in shorter TE and shorter scan time
	2020 Archer et al. [91]	Patients n = 18	SAVR n = 10 TAVR n = 8	−	+	−	Invasive peak pressure gradient and4D Flow CMR derived peak pressure gradient correlated well Prognostic advantage of 4D Flow CMR derived gradient vs. TTE
	2020 Callahan et al. [37]	Controls n = 6 Patients n = 8	Severe aortic stenosis	−	−	−	Combination of dual-VENC 4D Flow acquisition and spiral read-out offers increased velocity resolution and reduced scan time
Bicuspid aortic valve	2018 Bissell et al. [34]	Controls n = 30 Patients n = 60	Native BAV n = 30 Prior AVR n = 30	−	−	−	Normalisation of wall shear stress and rotational flow in patients with mechanical AVR or Ross procedure, but not those with bioprosthetic AV
	2019 Elbaz et al. [98]	Controls n = 34 Patients n = 57	BAV; stenotic and regurgitant valves	+	−	−	Kinetic energy, viscous energy loss rate and vorticity were reproducible in BAV patients Patients with severe AS showed highest levels of VELR and vorticity
	2019 Dux-Santoy et al. [96]	Controls n = 24 Patients n = 132	BAV n = 111; non-severe disease TAV with dilated arch n = 21	−	−	−	In-plane rotational flow, right/noncoronary BAV and systolic flow reversal ratio were predictors of aortic dilatation
	2020 Fatehi Hassanabad et al. [99]	Controls n = 11 Patients n = 32	BAV; stenotic and regurgitant valves	+	−	+	Larger pressure drop was observed in patients with > than moderate BAV stenosis
Tricuspid regurgitation	2008 Westenberg et al. [69]	Controls n = 10 Patients n = 20	Ischaemic cardiomyopathy with mitral regurgitation and/or TR	+	−	+	PCMR overestimated transtricuspid flow 4D Flow CMR showed strong agreement between MV and TV flow in patients with mitral regurgiatiton and/or TR
	2009 Roes et al. [25]	Controls n = 22 Patients n = 29	Ischaemic cardiomyopathy with valvular regurgitation	+	−	−	Agreement amongst net flow volume for all valves was excellent Good intra- and interobserver reliability for quantification of RF
	2018 Feneis et al. [9]	Patients n = 21	Isolated TR n = 6 Mitral regurgitation + TR n = 5 Isolated mitral regurgitaiton n = 10	+	−	+	Good correlation between PCMR and 4D Flow CMR quantification of regurgitation by direct and indirect methods
	2018 Driessen et al. [100]	Controls n = 21 Patients n = 67	RV pressure overload	+	+	+	Excellent concordance of effective TV flow vs. PCMR derived effective PV flow 4D Flow reclassified TR severity to a different grade vs. TTE grades
	2021 Juffermans et al. [26]	Patients = 64 Controls = 76	Various pathologies	+	−	−	Strong-to-excellent interobserver reliability for forward flow volume and net forward volume for all valves Moderate-to-excellent reliability for assessment of RF for all valves
Pulmonary regurgitation	2016 Chelu et al. [101]	Patients n = 52	Heterogenous group	+	−	+	Peak systolic PV velocity may be underestimated; this can be minimised by measuring the velocity where it appears to be the highest
	2019 Rizk et al. [103]	Controls n = 11 Patients n = 49	Tetralogy of Fallot n = 30 BAV n = 19	+	−	−	Severity of PR was proportional to peak diastolic WSS
	2020 Jacobs et al. [102]	Patients n = 34	Paediatric patients with repaired Tetralogy of Fallot	+	−	+	Pulmonary flow and aortic flow were most consistent at valve level RV ejection fraction was more reproducible by 4D Flow CMR vs. standard CMR RV volumes were mildly overestimated by 4D Flow CMR
	2021 Juffermans et al. [26]	Patients = 64 Controls = 76	Various pathologies	+	−	−	Strong-to-excellent interobserver reliability for forward flow volume and net forward volume for all valves Moderate-to-excellent reliability for assessment of RF for all valves

AR = aortic regurgitation; AS = aortic stenosis; AV = aortic valve; AVR = aortic valve replacement; BAV = bicuspid aortic valve; CHD = congenital heart disease; CMR = cardiovascular magnetic resonance; EROA = effective regurgitant orifice area; KE = kinetic energy; LV = left ventricle; LVEF = left ventricular ejection fraction; MV = mitral valve; MVR = mitral valve replacement; PCMR = phase contrast magnetic resonance; PISA = proximal isovelocity surface area; PR = pulmonary regurgitation; PV = pulmonary valve; RF = regurgitant fraction; RVol = regurgitant volume; RV = right ventricle; RVT = retrospective valve tracking; SAVR = surgical aortic valve replacement; SFT = semi-automated flow tracking; TAV = tricuspid aortic valve; TAVR = transcatheter aortic valve replacement; TKE = turbulent kinetic energy; TR = tricuspid regurgitation; TTE = transthoracic echocardiography; TV = tricuspid valve; VELR = viscous energy loss rate; VENC = velocity encoding; WSS = wall shear stress

**Fig. 1:**
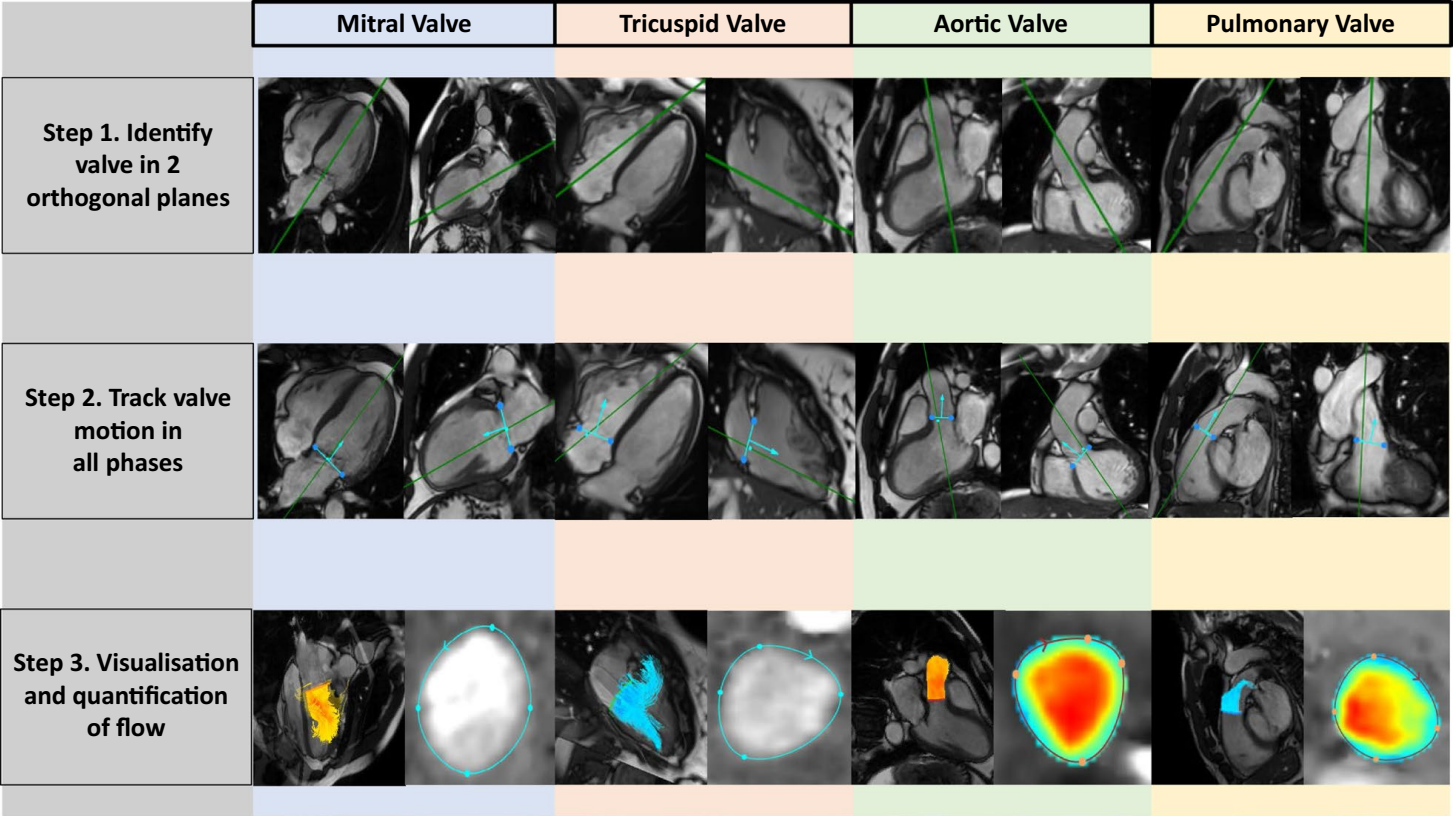
4D Flow CMR visualisation and quantification of valvular flow by retrospective valve tracking. Step 1. Identification of valve plane from cines acquired in 2 orthogonal planes. Step 2. Valve plane is tracked in all phases in the first view and cross-checked with the second view. Arrow allows confirmation of flow in the correct direction. Step 3. Visualisation of flow enables accurate quantification of flow in phase-contrast images

4D Flow CMR data are mostly analysed via retrospective valve tracking (RVT). RVT can be performed manually or by an automated process. In the case of the mitral valve, manual RVT is performed by first reformatting the mitral valve plane using the 4-chamber view and vertical long axis (VLA) of the LV. Manual placement of a line across the annulus in all the phases in the 4-chamber view marks the valve plane. This is cross-checked with the 2-chamber LV view to ensure correct positioning. This is subsequently performed manually in each phase. Once the valve is correctly tracked, a phase-contrast, valvular reformatted plane is created [69]. A study by Roes et al. showed good intra- and inter-observer reproducibility for this technique [25]. Automated valve tracking (Fig. 1) can be performed much more rapidly, and also with excellent intra- and inter-observer reproducibility [70]. Regurgitant flow can additionally be analysed by reformatting a plane, which is located above the annulus and is perpendicular to the regurgitant jet [26, 54]. Although most extensively studied in the setting of atrio-ventricular valves, retrospective valve tracking can also be applied to evaluation of semilunar valves. Recent studies showed, that aortic and pulmonary valve net forward flow and regurgitant flow can be directly quantified by RVT [26, 70]. Moreover, evaluation of valvular blood flow by automated RVT was shown to be reproducible and accurate for all valves, irrespective of scanner type and protocol [26].

Different studies have explored different methods of mitral regurgitant volume quantification by 4D Flow CMR. The main techniques included the indirect method (mitral regurgitant volume = 4D Flow CMR mitral inflow volume − 4D Flow CMR aortic outflow volume [Mitral Regurgitation_MVAV_], direct method, which quantified mitral regurgitation directly from 4D Flow phase contrast images and the 4D-CMR PISA method. These techniques are described below:

1. 4D flow CMR indirect method (MR_MVAV_)

A study by Fidock et al., showed that mitral regurgitatant volume assessed by the 4D Flow CMR derived aortic outflow-mitral inflow calculation, correlated well with standard PCMR in primary mitral regurgitation, secondary mitral regurgitation and even in patients with mitral valve replacement (MVR); and had the highest level of concordance with the standard PCMR measurements [54]. Another study of 54 patients with mitral regurgitation secondary to mitral valve prolapse (MVP), compared RVol and RF by 4D Flow CMR direct jet quantification and indirectly by 4D Flow derived difference in aortic SV and mitral inflow. The direct jet interrogation technique was shown to have a lower inter- and intra-technique consistency than the 4D Flow CMR indirect method. The indirect method agreed well with PCMR, whereas the direct technique yielded much lower regurgitant volumes. This was felt to be secondary to the physiology of mitral regurgitant jets, which tend to be multiple and eccentric in nature [42].

**Fig. 2:**
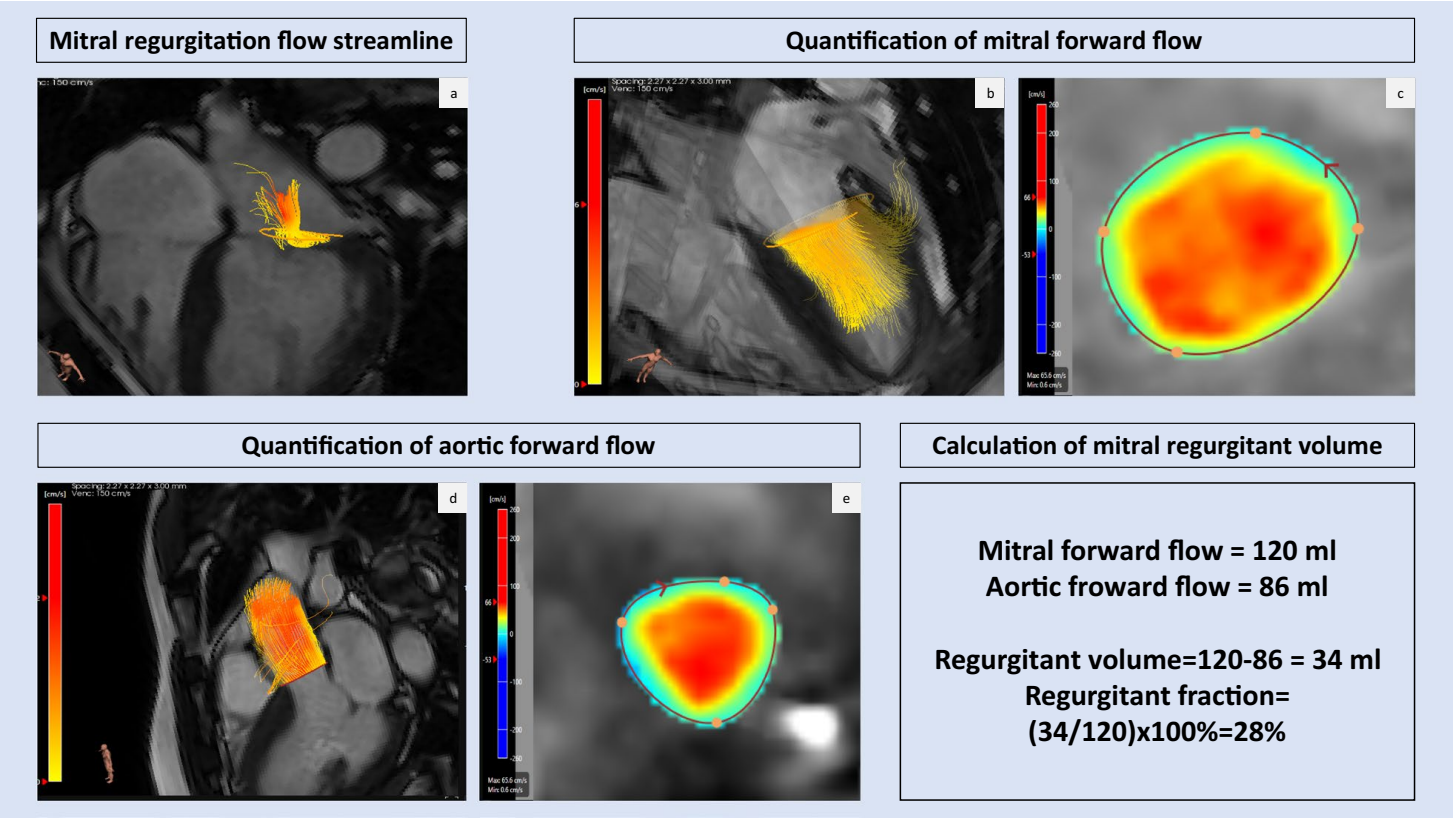
4D Flow CMR assessment of mitral regurgitation. **a** Shows four-dimensional mitral regurgitation flow streamline. **b** Demonstrates mitral forward flow visualised by 4D Flow CMR and **c** quantification of mitral forward flow by phase-contrast image obtained from 4D Flow CMR. **d** Shows aortic forward flow and **e** quantification of aortic forward flow by phase-contrast image obtained by 4D Flow CMR

Although direct quantification at jet level can be reliable and accurate, even in the presence of multiple and eccentric regurgitant jets [41], the indirect method offers an advantage, especially in cases of very complex regurgitant jets, where the direct jet method can be challenging to perform and labour intensive [40, 54]. An example of 4D Flow CMR indirect assessment of moderate mitral regurgitation is shown in Fig. 2.

2. Direct mitral regurgitation quantification

An early study by Roes et al. showed that net flow through all valves can be assessed accurately with RVT and demonstrated good intra- and inter-observer consistency for regurgitant fraction in patients with mitral regurgitation [25]. A more recent study by Kamphuis et al. compared net forward flow and regurgitant fraction by automated RVT versus manual valve tracking, and demonstrated that automated RVT can be performed much more rapidly than manual valve tracking, but also with high intra- and inter-observer reproducibility for RF [70].

A small study of patients with ischaemic heart disease assessing LV diastolic parameters, found significantly lower transmitral flow rates and higher mitral valve RF when compared to PCMR [71]. Similarly, a study of healthy subjects and patients with mitral regurgitation showed that in healthy subjects, mitral valve flow was overestimated by 15% when assessed by PCMR [69]. However, when compared to TTE, the results were somewhat variable. Brandst et al. found an excellent correlation between 4D Flow CMR and TTE versus PCMR and TTE [71], whereas a study by Marsan et al. found that 2-dimensional (2D) TTE significantly underestimated mitral RVol in patients with functional mitral regurgitation as compared to 4D Flow CMR [72]. All studies mentioned so far mainly used RVT at the valve level to quantify mitral regurgitation.

Feneis et al. utilised direct jet analysis as well as the indirect method in the assessment of 21 patients with mitral regurgitation and/or TR and compared the results to conventional CMR. Direct jet assessment involved examination of the regurgitant flow at various anatomical locations, beginning at the valve plane and then with at least 5 mm intervals. Areas of signal dephasing and aliasing velocity were avoided. The authors showed, that there was a good correlation between the measurements for those 2 modalities, for both, the direct and the indirect method. The intraobserver and interobserver reproducibility also proved very good to excellent for the direct jet approach [9].

Direct jet interrogation was also used in a recent study by Fidock et al. of 35 patients with primary mitral regurgitation, secondary mitral regurgitation and MVR. Fidock et al. found that in cases of primary mitral regurgitation, direct jet interrogation overestimated mitral regurgitant volumes; this was not the case for secondary mitral regurgitation or MVR patients. The authors proposed that the inconsistency in delineating the analysis plane was most likely responsible for this finding. The concordance of results in secondary mitral regurgitation was most likely related to the central regurgitant jet and the particular cohort of patients in this study, as most patients with secondary mitral regurgitation had only a mild degree of regurgitation [54].

A recent study by Blanken et al. quantified mitral regurgitation by semi-automated flow tracking (SFT) and compared it to semi-automated valve tracking. In this retrospective study of 30 patients, the authors showed that flow tracking allowed superior assessment of mitral regurgitation, especially in cases of severe mitral regurgitation. This study showed that RVol assessed by flow tracking was higher than the volume quantified by valve tracking and it correlated better with the RVol assessed by the standard method (LV SV—aortic outflow). Mitral regurgitation volume was underestimated by the valve tracking technique in cases of severe mitral regurgitation. The interobserver reproducibility was superior for SFT versus semi-automated valve tracking. The authors proposed that the superiority of flow tracking may be related to the enhanced precision of quantification of flow by relocating the analysis plane above the annulus and thus avoiding areas of turbulence and dephasing; and improved differentiation of the mitral regurgitant jet and aortic forward flow. The authors noted, however, that there were several limitations, which may have been responsible for the findings, including different aetiologies of mitral regurgitation in the different valve severity groups [73].

3. PISA method

Although this is not an actual 4D Flow method per se, Gorodisky et al. showed that CMR 4D-proximal isovelocity surface area (PISA) is feasible as a surrogate marker of mitral regurgitant volume quantification. In this method, 3D flow vectors are obtained from each 3 mm slice between the mitral valve annulus and the LV apex. Although the analysis is performed by automated software, the appropriate slices and time frames need to be chosen manually. CMR 4D-PISA excludes geometric assumptions that are invariably made by echocardiography with this method. When compared to TTE-PISA, the CMR-PISA was smaller. TTE-PISA frequently overestimates flow, as it is obtained at a single time-point and does not take into account variation of flow during systole. The shape of CMR-PISA was also noted to be a hemi-ellipsoid in contrast to the hemisphere on which the TTE-PISA assumptions are based. The authors suggested, that flow magnitude could be measured accurately, as 3D velocity encoding allowed for the true flow to be measured in each voxel, diminishing the errors caused by the angle between the flow direction and the imaging plane. However, one disadvantage of CMR-PISA method is the possibility of inaccurate localisation of the vena contracta, which can occur in some cases [74]. Of note, the accuracy of flow assessment may be influenced by the sequence used. A recent study showed, that accelerated echo-planar imaging sequence may lead to errors in flow and velocity measurements in certain cases [75].

Finally, novel markers such kinetic energy (KE) mapping by 4D Flow CMR can also be utilised to aid the assessment of mitral regurgitation [21]. One study showed that peak KE levels in the late diastolic period did not decline after mitral valve surgery, suggesting persistence of pathological blood flow after an intervention [76].

As 4D Flow CMR offers a lot of advantages in quantification of mitral regurgitation, it may help to correlate long-term outcomes of patients with significant regurgitation according to different thresholds of severity, and clarify what RVol and RF are associated with adverse LV remodelling and may benefit from earlier surgical treatment [9]. While there are advantages and disadvantages associated with all the aforementioned techniques, the indirect method (*MR*_*MVAV*_) has been shown to be most accurate and reproducible.

#### Mitral stenosis

**Fig. 3:**
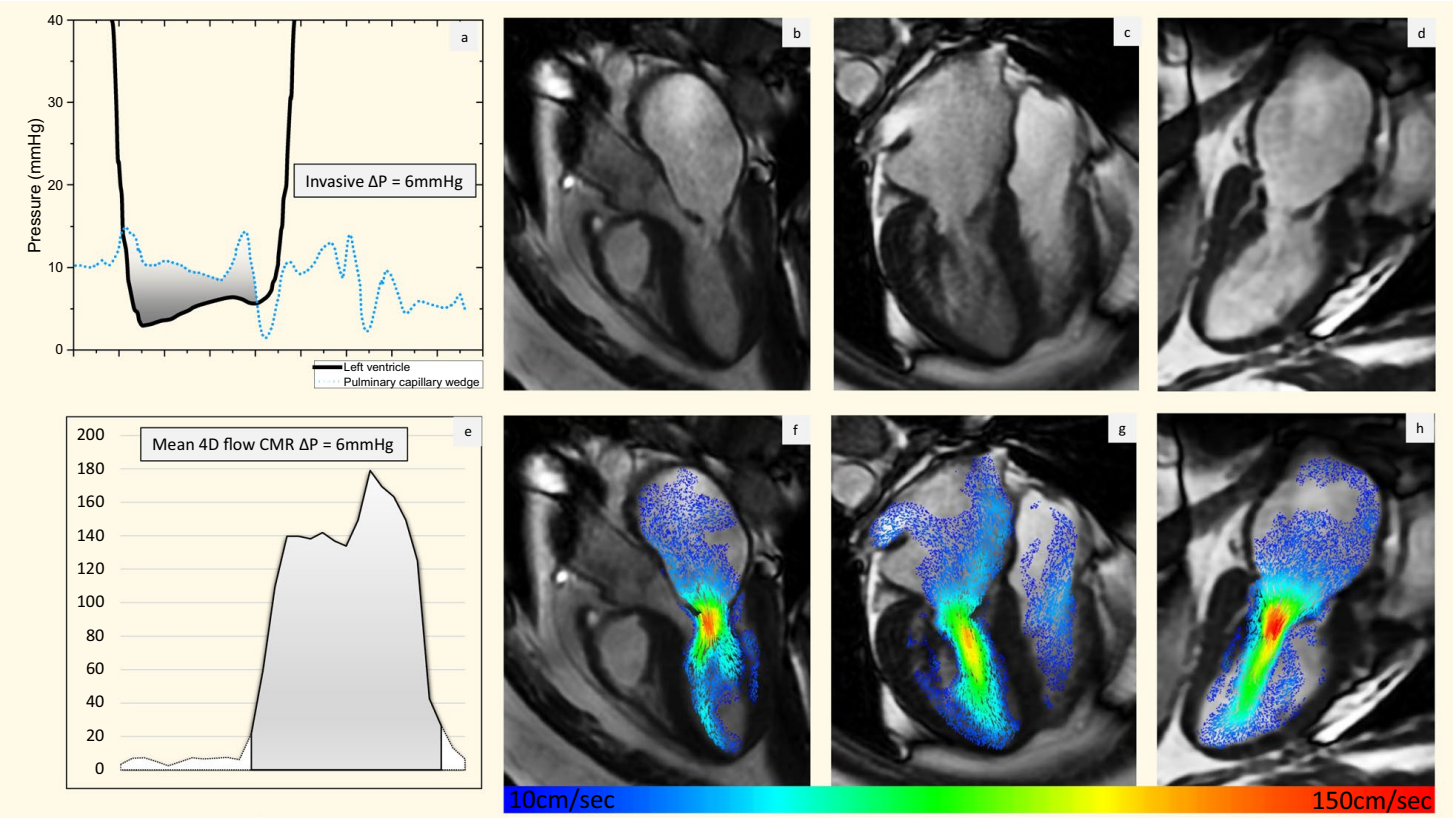
Invasive and 4D Flow CMR assessment of moderate mitral stenosis. **a** Demonstrates invasive assessment of mitral stenosis with simultaneous recording of pulmonary artery wedge pressure and left ventricular pressure. The mean pressure gradient by this method was 6 mmHg. **b**–**d** CMR left ventricular outflow tract (LVOT) view, 4-chamber view and vertical long axis view, demonstrating reduced opening and thickening of the mitral valve leaflets and left atrial dilatation. **e** demonstrates quantification of peak velocity in the red areas of peak velocity zone on **f**–**h**. **f**–**h** In-plane velocities are superimposed on the above images, demonstrating increased forward flow velocity through the mitral valve

Although there are no studies of 4D Flow CMR in mitral stenosis, we have noted in our practice that mitral stenosis severity assessment by 4D Flow CMR-derived mean pressure drop correlates well with invasive measurement and mitral valve planimetry by CMR [77]. Mitral valve planimetry by TTE is the reference-standard in the assessment of mitral stenosis severity [1] and a good correlation between mitral valve planimetry by CMR and TTE has been previously shown [78]. An example of 4D Flow CMR and invasive assessment of moderate mitral stenosis is shown in Fig. 3.

#### Aortic regurgitation

Evaluation of aortic regurgitation (AR) is primarily performed by TTE, and additionally with transesophageal echocardiography (TEE) and CMR in selected cases. TTE enables quantification of the severity of AR and its effect on LV cavity size and function [79], with indications for surgical timing in AR based on patient symptoms plus these aforementioned parameters [3]. Accurate assessment of regurgitation severity is crucial to allow optimal timing of intervention before irreversible LV remodelling occurs. As discussed, CMR provides the reference-standard evaluation of LV volume and function [14], with studies also showing that aortic RVol quantification by CMR correlates better with clinical outcomes than volumetric TTE measurements [80], and also has better prognostication than CMR assessment of LV volumes alone [81].

Assessment of AR by PCMR requires through-plane velocity encoded images. Although it is recommended to plan these images in a plane located 5 mm above the valve [82] and perpendicular to the vessel [83], in clinical practice this is frequently adjusted, as presence of turbulence and vortex formation secondary to eccentric jets may lead to inaccurate quantification of flow at this level and thus images are acquired at a level where flow appears more laminal. Care needs to be taken not to place the analysis plane too distally in the aorta, as this could lead to underestimation of regurgitation [84].

There are a number of advantages of 4D Flow CMR in the setting of AR when compared to PCMR. Functional and flow data are obtained from a single, free-breathing acquisition [85]. Furthermore, the annulus can be tracked accurately during the entire cardiac cycle [86].

In dilated aorta, we routinely see circular flow which makes it very challenging to quantify true forward flow through the vessel. In these challenging cases, 4D Flow allows to identify a plane with the most laminar flow in the ascending aorta to quantify forward flow. Therefore, 4D Flow CMR could potentially improve the accuracy of quantification of flow in the presence of a dilated aorta [79].

It is usually recommended, however to still cautiously segment the flow once a reformatted phase contrast plane is generated using the 4D Flow dataset.

**Fig. 4:**
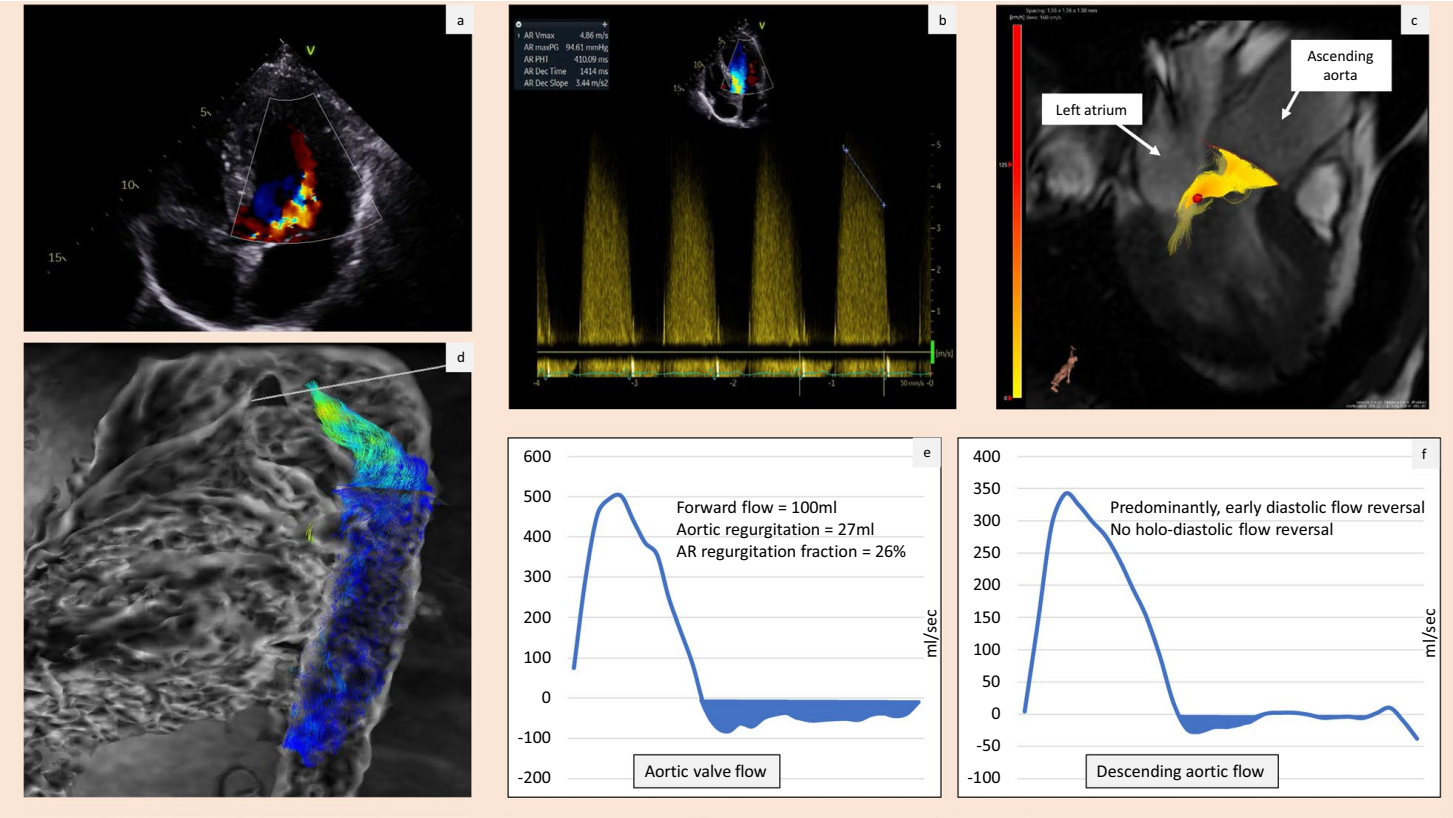
TTE and 4D Flow CMR assessment of moderate aortic regurgitation. **a** 4-chamber colour Doppler transthoracic echo (TTE) demonstrating aortic regurgitation jet and **b** TTE continuous wave Doppler demonstrating pressure half time consistent with moderate aortic regurgitation. **c** is a four-dimensional flow streamline visualisation of aortic regurgitation in left ventricular diastole. **d** is pathline visualization of flow reversal in the early diastole in the descending aorta. **e** is a reformatted **c** at aortic valve level flow quantification. **f** is the reformatted plane at the descending aorta level flow quantification

A number of studies evaluated the feasibility of 4D Flow CMR in the assessment of AR (Table 2). A study by Ewe et al. of 32 patients compared RVol obtained from 2 and 3D TTE with 4D Flow CMR. This study demonstrated good correlation and agreement between AR severity assessed by 3D TTE and 4D Flow CMR; 2D TTE was less reliable. The correlation remained strong even in the presence of eccentric jets [86]. Another study of 54 patients compared 4D Flow CMR values against TTE. The concordance of these 2 methods was good, and more importantly 4D Flow CMR showed 100% sensitivity and 98% specificity for detection of more than mild AR [85]. A recent study by Alvarez et al. published in 2020 directly compared AR severity quantification by 4D Flow CMR versus PCMR. It evaluated 34 patients with AR RF of at least 5% as assessed by PCMR. The authors found an excellent agreement between the two techniques in terms of forward flow, regurgitant flow and RF [79]. An example of TTE and 4D Flow CMR assessment of moderate AR is shown in Fig. 4.

#### Aortic stenosis

Aortic stenosis (AS) is an increasingly common valvular pathology in the developed world [1]. Similar to other valvular lesions, the indications for intervention incorporate symptoms as well as timely and accurate assessment of AS severity [3]. Although TTE is the first-line investigation in most patients, PCMR provides a detailed and non-invasive option in challenging cases, where the severity of AS remains unclear. For accurate assessment of peak velocity, it is crucial to perform the through-plane velocity encoding by positioning the imaging plane parallel to the aortic valve and in a plane that corresponds to the highest velocity, characterised by turbulence in long-axis aortic valve cine views [82]. Appropriate VENC also needs to be chosen to avoid errors in peak velocity measurements [83]. Additionally, CMR can precisely assess LV function [6] and detect the presence and extent of fibrosis [46, 87, 88].

4D Flow CMR provides not only additional information related to valve severity and its impact on the LV, but it allows evaluation of consequences of pathological flow through the valve on the ascending aorta [22, 89]. It also has the potential to accurately assess peak velocities, even in the presence of multiple or eccentric jets [21]. Studies which evaluated 4D Flow CMR in AS are summarised in Table 2.

A study published by Garcia et al. proposed the use of 4D Flow jet shear layer detection method for measurement of the effective orifice area (EOA) in AS. This was validated against results based on the continuity equation method with values obtained from PCMR. 4D Flow CMR offered the advantage of 3D projection of the vena contracta, and thus allowed a more accurate localisation [90]. A recent study by Archer et al. assessed 18 patients with severe AS with TTE and 4D Flow CMR pre- and post-intervention. Patients undergoing transcatheter aortic valve replacement (TAVR) also underwent invasive pressure measurements. The authors showed that there was a good correlation between the invasive peak pressure gradient and the 4D Flow derived gradient. This study also demonstrated the prognostic advantage of 4D Flow derived pressure gradient versus TTE as shown by LV remodelling after the intervention [91].

**Fig. 5:**
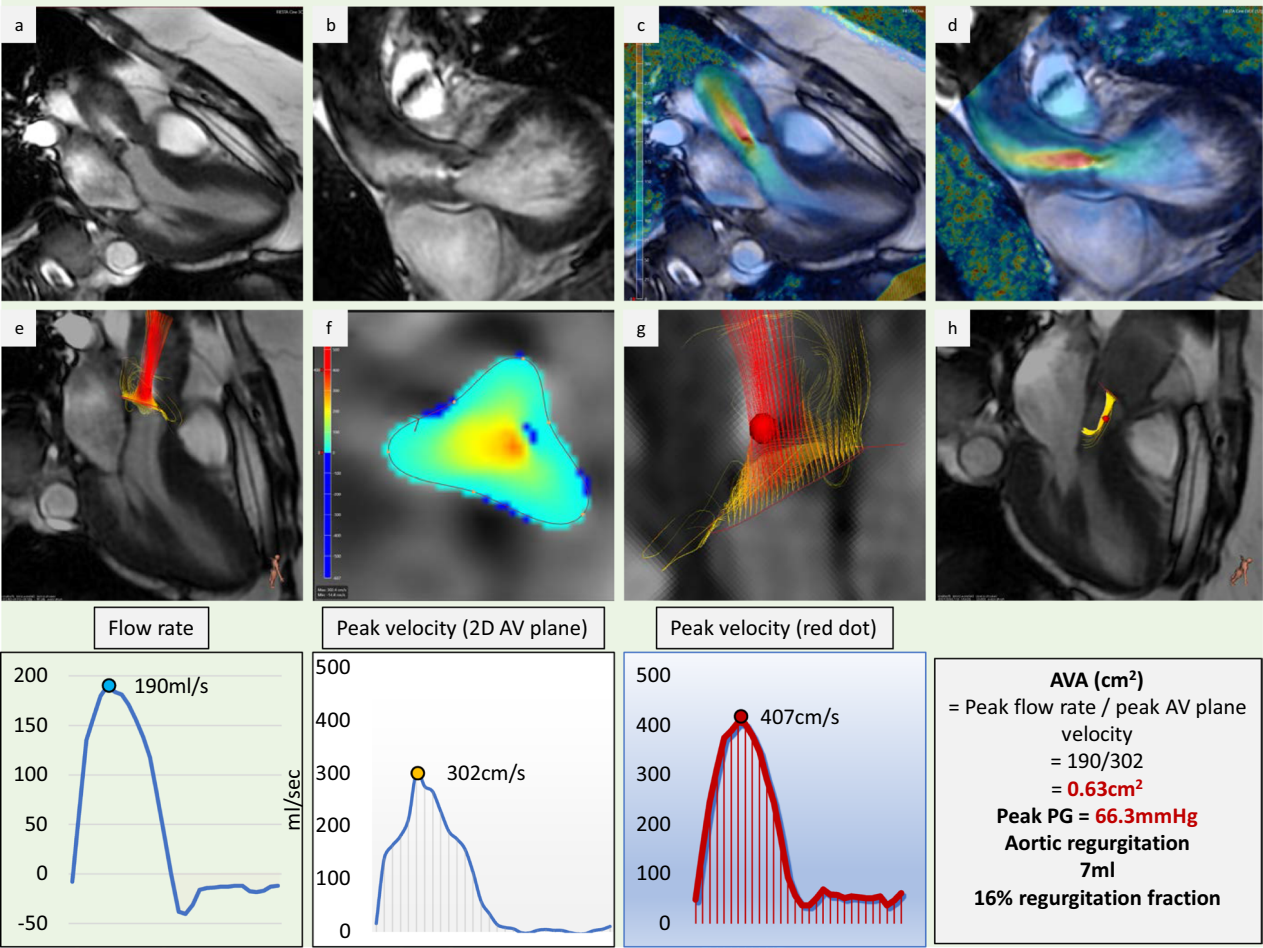
4D Flow CMR assessment of severe aortic stenosis. **a** is a sagittal LVOT view and **b** coronal LVOT view demonstrating restrictive aortic valve opening with dephasing artefact demonstrating higher velocities through a narrow orifice. **c** and **d** further demonstrate increased velocity by superimposing in-plane velocity overlay onto the sagittal and coronal LVOT view. **e** shows four-dimensional flow streamline tracing through the aortic valve. **f** is a reformatted phase-contrast plane through the aortic valve demonstrating quantification of aortic valve forward flow and peak velocity. **g** is a zoomed in images of **e** demonstrating where the peak velocity through the aortic valve is (red ball). **h** Shows streamline visualisation of aortic regurgitation into the outflow tract

As 4D-flow acquisitions can be time-consuming, several alternative k-space acquisition methods have been studied to optimise scan time. An in-vitro and in-vivo study of spiral k-space readout in patients with AS demonstrated shortening of echo time (TE) and the overall scan time. Peak velocity acquired with a spiral readout also correlated better with TTE Doppler measurements, than in the standard Cartesian acquisition [92]. A recent study by Callahan et al. evaluated dual VENC acquisition with a spiral readout. The authors showed that the spiral readout resulted in a reduction of flow-related artefacts due to lower TE, while dual VENC acquisition allowed better velocity resolution and reduction of noise [37]. An example of 4D Flow CMR assessment of severe AS is shown in Fig. 5.

A novel marker that may be evaluated by 4D Flow imaging is turbulent kinetic energy (TKE). Turbulent blood flow in the presence of valvular lesions, such as AS, leads to energy loss by heat release, which can be quantified by TKE [21]. A small study of 4 healthy subjects and 14 AS patients, showed that there was a good agreement between the peak total TKE in the ascending aorta and pressure loss index based on standard assessment. Patients with AS also demonstrated much larger TKE in the ascending aorta as compared to healthy controls [93]. At present, TKE is only available as a research tool.

Low-flow low-gradient AS frequently poses a diagnostic challenge in clinical practice. Whereas there is a considerable amount of data supporting the use of 4D Flow CMR in the setting of high-gradient AS, data regarding 4D Flow CMR in the setting of low-flow low-gradient AS are scarce. Comprehensive assessment of advanced markers such as turbulent kinetic energy may be helpful in this cohort [94]. One study suggested that 4D Flow CMR-based turbulence production method of pressure drop assessment may be particularly useful in quantifying severity in low flow condition, such as paradoxical low-flow low-gradient AS, as it does not presume negligible viscosity [95].

Several studies evaluated bicuspid aortic valve disease with 4D Flow CMR and the associated changes in flow patterns. The altered haemodynamics have an adverse effect on the aorta and therefore may explain why BAV is frequently associated with an aortopathy [34, 96]. Although TTE and PCMR provide a comprehensive assessment of BAV, 4D Flow CMR offers a number of advantages in this particular pathology. These are briefly described below, as 4D Flow CMR assessment of pathological aortic flow patterns in BAV is beyond the scope of this review.

4D Flow CMR allows visualisation and interrogation of abnormal flow patterns in BAV, which may result in altered wall shear stress (WSS) and subsequently aortic dilation in certain cases [97]. Measurements of TKE losses, amongst other advanced parameters, may help in risk stratification [94]. A study of 111 patients with BAV demonstrated that in-plane rotational flow, systolic flow reversal ratio and right/non-coronary cusp fusion subtype were predictors of aortic dilatation, suggesting the need for closer monitoring in these subgroups [96].

Elbaz and colleagues proposed the use of a 4D-virtual catheter for the assessment of intra-aortic haemodynamics in patients with BAV. The authors showed that markers such as KE, viscous energy loss rate (VELR) and vorticity were reproducible. In this study, patients with severe AS demonstrated the highest levels of VELR and vorticity [98]. Bissell et al. examined changes in flow patterns following intervention in BAV patients. Interestingly, the authors showed that patients with mechanical aortic valve replacement (AVR) or Ross procedure had normalisation of in-plane wall shear stress and rotational flow versus those with bioprosthetic AV, who didn’t demonstrate a similar effect [34]. Pressure drop mapping is another parameter that can be measured by 4D Flow CMR in patients with BAV. Although pressure drop is present in healthy controls, a significantly higher pressure drop was observed in patients with BAV; and there was an association between pressure drop and severity of stenosis [99].

#### Tricuspid regurgitation

Tricuspid valve is often difficult to visualise by TTE, which renders the evaluation of tricuspid regurgitation (TR) potentially inaccurate with poor reproducibility. Similar to the mitral valve, direct assessment of TR by PCMR is imprecise due to through-plane valve motion. Although indirect assessment of TR volume by subtracting pulmonary valve (PV) outflow from RV SV is routinely used in clinical practice, it is also limited by potential errors, mainly in stroke volume calculation [100]. Accurate assessment is crucial, as the severity of TR guides treatment decisions [9].

There are only a small number of studies, that have evaluated the tricuspid valve by 4D Flow CMR (Table 2). Techniques used to quantify TR volume and fraction included the direct and indirect method, similar to assessment of mitral regurgitant severity [9, 25, 69]. Interestingly, the agreement between direct and indirect assessment by 4D Flow CMR was shown to be better for TR than mitral regurgitation. Interobserver agreement also compared favourably in cases of TR versus mitral regurgitation. This can possibly be explained by the nature of TR jet, which is characteristically more uniform and laminar [9].

Westenberg and colleagues showed, that not only there was a good agreement between 4D Flow CMR quantified tricuspid forward flow volume and aortic systolic flow volume in healthy subjects, but also that this agreement was better for 4D Flow CMR than PCMR. There was also a good agreement between mitral valve and tricuspid valve flow in both, the healthy subjects and patients with regurgitation; both of which can be overestimated with PCMR. 4D Flow CMR may therefore be particularly useful in the assessment of TR volume and fraction, as similar to mitral regurgitation, PCMR does not account for valve annulus through-plane motion during systole. The SNR was, however, lower with 4D Flow imaging, as acceleration techniques had to be utilised to reduce scan time. Although theoretically this could reduce the accuracy of results due to lower image quality, the authors felt that in this case SNR was adequate for precise flow assessment [69] Roes et al. also demonstrated high agreement between net flow volumes across all valves, including in patients with one or more regurgitant lesion [25].

**Fig. 6:**
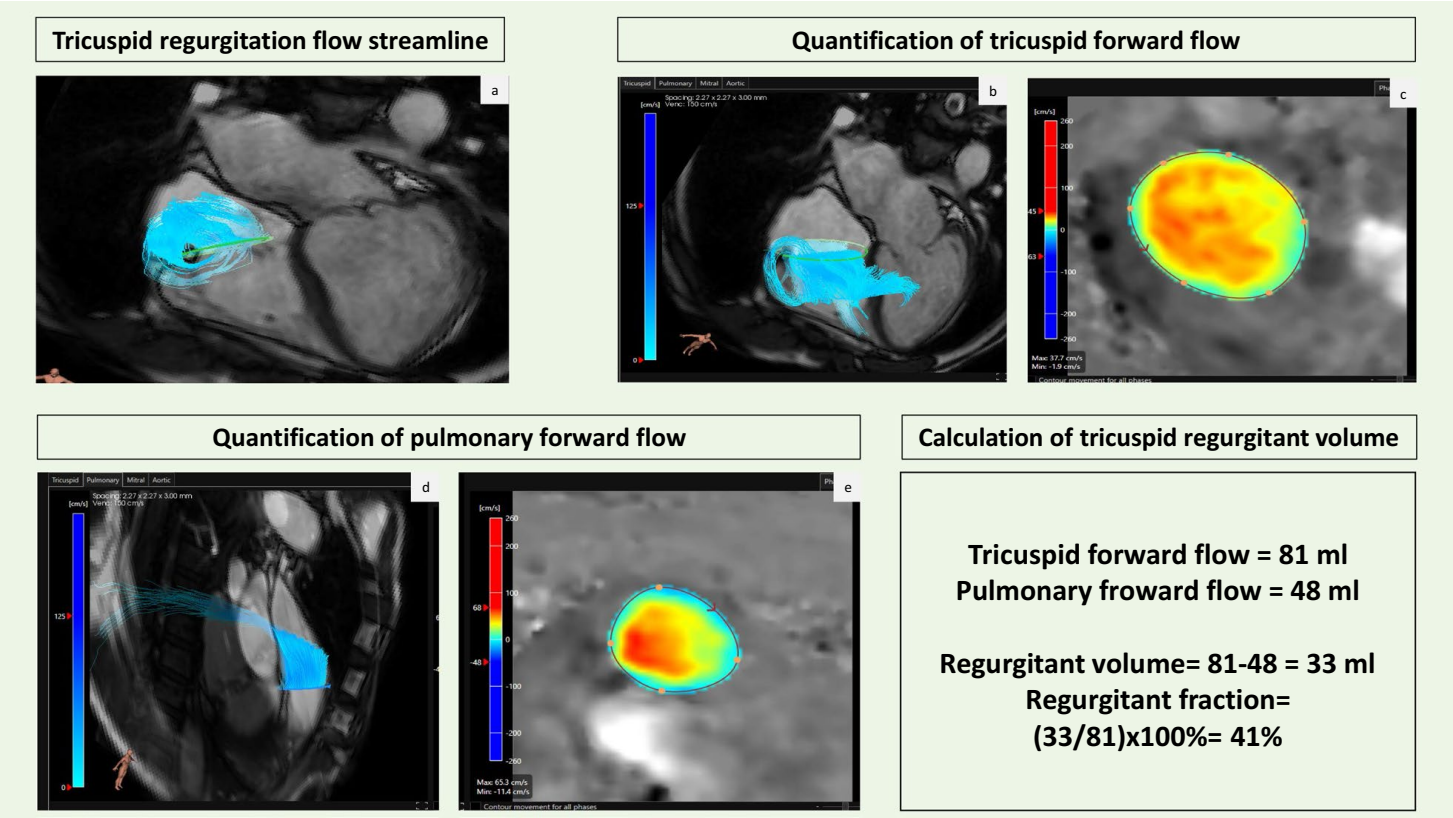
4D Flow CMR assessment of tricuspid regurgitation. **a** shows 4D flow streamline of tricuspid regurgitation. **b** demonstrates tricuspid forward flow visualised by 4D Flow CMR and **c** quantification of tricuspid forward flow by phase-contrast image obtained by 4D Flow CMR. **d** Shows pulmonary forward flow and **e** quantification of pulmonary forward flow by phase-contrast image obtained by 4D Flow CMR

TR in the setting of congenital heart disease (CHD) provides further diagnostic challenges. A study of 21 healthy subjects and 67 patients with RV pressure overload secondary to pulmonary hypertension, pulmonary stenosis, tetralogy of Fallot with PV stenosis or systemic RV found, that the effective flow volume across the TV quantified by 4D Flow CMR and the effective pulmonic valve flow assessed by PCMR correlated well. There was discordance between TR severity grades in almost 40% of cases when compared to TTE. Despite the difficult anatomy in this cohort, 4D Flow CMR was shown to have high reproducibility [100]. An example of 4D Flow CMR assessment of moderate TR is shown in Fig. 6.

#### Pulmonary regurgitation

The pulmonary valve is less often assessed in routine adult cardiology practice. However, pulmonary regurgitation (PR) is common in the CHD population and a detailed assessment of the PV is standard practice. Although PCMR is a well-established method of PR evaluation, the acquisition may be difficult and requires careful planning. It is recommended that velocity encoded imaging plane should be placed about 5 mm above the valve [82] and positioned perpendicular to the vessel [83]. However, similar to AR quantification, this is frequently optimised in clinical practice to avoid areas of turbulence and dephasing.

4D Flow CMR offers many advantages in the assessment of PR, including a relatively simple acquisition and direct visualisation of the regurgitant flow [101]. Table 2 summarizes 4D Flow CMR studies in PR.

**Fig. 7:**
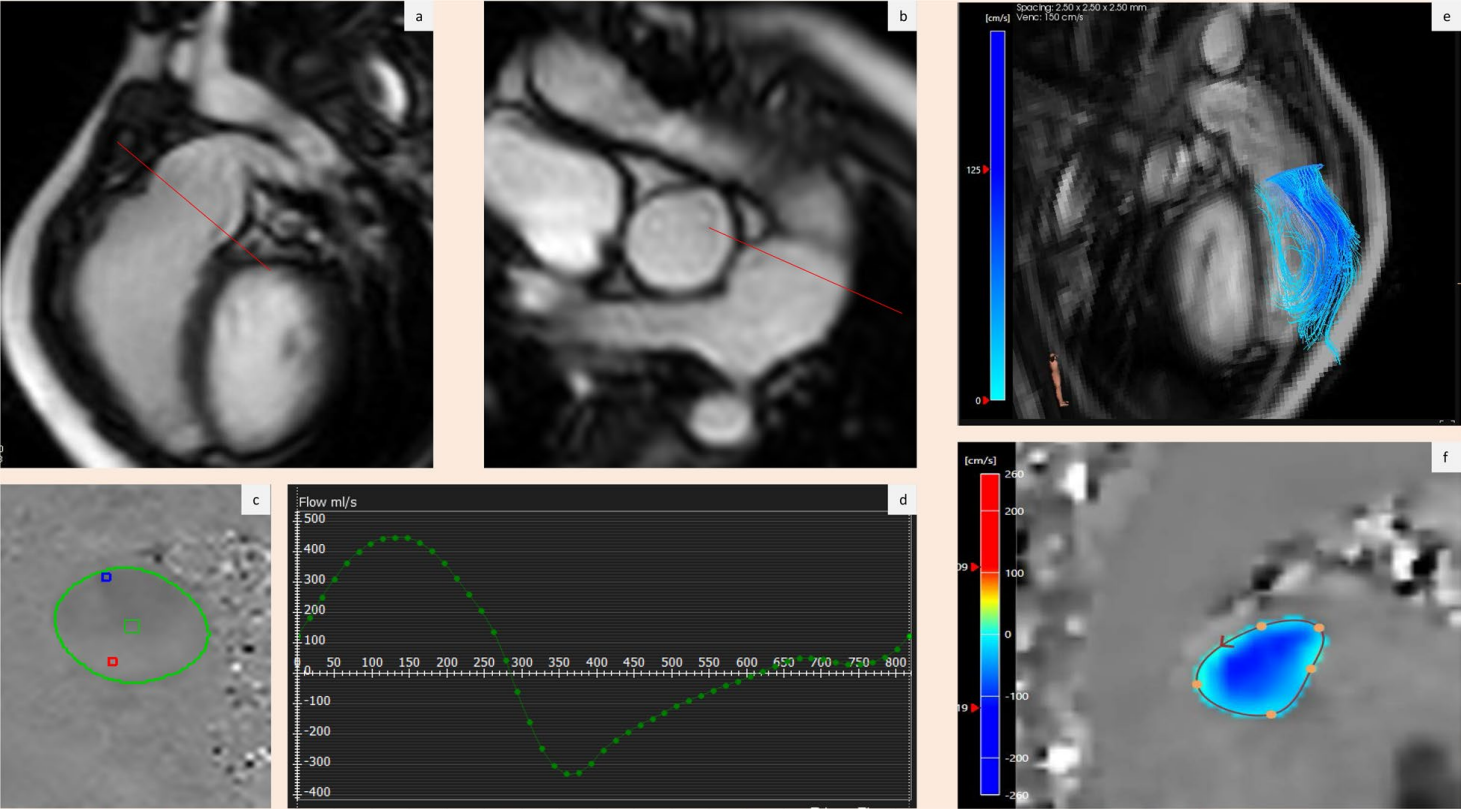
Phase Contrast CMR and 4D Flow CMR assessment of pulmonary regurgitation post tetralogy of Fallot repair. **a** and **b** Show sagittal and coronal right ventricular outflow tract views used for planning of pulmonary through-plane flow. Red line demarcates the analysis plane. **c** and **d** Show quantifications of pulmonary flow by PCMR revealing significant regurgitant flow. **e** Shows significant pulmonary regurgitation by 4D Flow CMR. **f** Demonstrates quantification of pulmonary regurgitant flow by 4D Flow CMR

The main application of 4D Flow in PR is in patients with repaired tetralogy of Fallot [51]. In a recent publication, Jacobs and colleagues evaluated PR in a paediatric population by measuring pulmonary net flow (PNF). The closest concordance of PNF by 4D Flow CMR with aortic valve flow was found to be at the valve level. PV forward flow and RV stroke volume demonstrated more than moderate agreement. This study also found, that there was a slight overestimation of RV volumes and function by 4D Flow CMR as compared with PCMR. Importantly, the 4D Flow acquisition was significantly shorter than the acquisition of a complete, conventional PCMR scan protocol, which is crucial in a paediatric population [102]. A prospective study of 52 adult patients, mostly with various CHD pathologies demonstrated, that pulmonary flow and PR can be reliably assessed via 4D Flow CMR, although peak systolic velocity can be underestimated as compared to PCMR. This error can be minimised by measuring velocity in the pulmonary artery where it is likely to be the highest [101]. Comprehensive evaluation of PR also includes the assessment of peak diastolic WSS, which was shown to be associated with severity of PR [103]. An example of 4D Flow CMR assessment of significant PR in the setting of repaired tetralogy of Fallot is shown in Fig. 7.

### Limitations

Limited spatial and temporal resolution are significant drawbacks of 4D Flow CMR [16]. These can lead to underestimation of high-velocity regurgitant jets [9]. Long scan times may also limit the utility of this technique in a number of settings such as claustrophobic or unwell patients who may not tolerate such a lengthy scan [29, 51]. Newer acceleration techniques, however, enable faster acquisition without a compromise in terms of spatial or temporal resolution. Labour-intensive post-processing further limits the clinical applicability of 4D Flow [29]. Similar to PCMR, phase offset errors can cause substantial artefacts and need to be corrected. Although effects of eddy currents cannot be entirely removed, they can be minimised [16].

### Current status and future perspective

In routine clinical practice, an experienced 4D Flow user can complete quantification of aortic and pulmonary flow in about 5 min. Careful segmentation of mitral valve flow adds 5–10 min. If direct jet approach is used, it adds a few minutes to the post-processing time.

Despite all the developments of 4D Flow CMR in the recent years, it has not become a routine component of CMR protocols. Expensive analysis software, time consuming post-processing and expert analysis required all hinder adoption of 4D Flow in everyday CMR practice. Development of an approachable, user-friendly software could encourage more imaging specialists to train in this novel technique, increasing its clinical utility.

## Conclusions

Assessment of VHD by 4D Flow CMR is precise and reproducible. Although still a relatively novel technique and not routinely employed in contemporary clinical practice, it has the potential to become the new reference-standard method for the evaluation of valvular lesions. It overcomes a lot of the constraints of other imaging modalities, including TTE, TEE and PCMR. With new acceleration techniques and developments in automated post-processing methods, scan times and post-processing times are likely to be substantially shortened in the future, making this technique much more clinically applicable.

## Data Availability

All data presented in this review are available via online search using Ovid MEDLINE database and Google Scholar.

## Abbreviations

2D: Two-dimensional
3D: Three-dimensional
4D: Four-dimensional
AF: Atrial fibrillation
Ao: Aortic flow
AR: Aortic regurgitation
AS: Aortic stenosis
AV: Aortic valve
AVR: Aortic valve replacement
BAV: Bicuspid aortic valve
CHD: Congenital heart disease
CMR: Cardiovascular magnetic resonance
C-SENSE: Compressed sensitivity encoding
ECG: Electrocardiogram
ECG: Effective orifice area
EROA: Effective regurgitant orifice area
KE: Kinetic energy
Kt-BLAST: Kt broad linear speed up technique
LA: Left atrium/left atrial
LV: Left ventricle/left ventricular
LVEF: Left ventricular ejection fraction
LVOT: Left ventricular outflow tract
MIP: Maximum intensity projection
MRI: Magnetic resonance imaging
MV: Mitral valve
MVP: Mitral valve prolapse
MVR: Mitral valve replacement
PCMR: Phase contrast magnetic resonance
PISA: Proximal isovelocity surface area
PNF: Pulmonary net flow
PR: Pulmonary regurgitation
PROUD: Prospective undersampling in multiple dimensions
PV: Pulmonary valve
RF: Regurgitant fraction
RV: Right ventricle/right ventricular
RVol: Regurgitant volume
RVT: Retrospective valve tracking
SAVR: Surgical aortic valve replacement
SFT: Semi-automated flow tracking
SNR: Signal-to-noise ratio
SV: Stroke volume
TAVR: Transcatheter aortic valve replacement
TE: Echo time
TEE: Transesophageal echocardiography
TKE: Turbulent kinetic energy
TR: Tricuspid regurgitation
TTE: Transthoracic echocardiography
TV: Tricuspid valve
VELR: Viscous energy loss rate
VENC: Velocity encoding
VHD: Valvular heart disease
VLA: Ventricular long axis
WSS: Wall shear stress
